# Delay discounting and anxiety: a systematic review on current evidence for clinical and non-clinical population

**DOI:** 10.3389/fpsyg.2025.1645442

**Published:** 2025-10-19

**Authors:** Miguel Domínguez Rojas, Carlos Velo Higueras

**Affiliations:** Universidad Europea de Valencia, Faculty of Health Sciences, Valencia, Spain

**Keywords:** delay of gratification, anxiety, delay discounting, temporal discounting, systematic review

## Abstract

**Introduction:**

Delay discounting (DD) is a psychological process that involves the tendency to prefer immediate rewards over delayed rewards, even if the latter are greater. The study of this process has been approached from different conceptualisations, including delay discounting, intertemporal choice and temporal discounting. The study of this construct began in the field of economics, but has subsequently been applied to various fields of psychology. There seems to be a generalised tendency among people with various pathologies to prefer immediate and smaller rewards to larger but delayed ones, although this tendency is not clear in the case of people with anxiety.

**Method:**

To study the relationship between anxiety and DD, a systematic review was carried out in accordance with the PRISMA 2020 guidelines, including 25 studies with a total sample of 12,728 subjects from the clinical and general population.

**Results:**

The results point to a positive relationship between anxiety and DD as found in most studies in both clinical and general populations. Other studies have provided some interesting nuances on this relationship. Only two studies have found a negative relationship between DD and anxiety.

**Discussion and conclusion:**

The analysis of our review suggests a positive relationship between DD and anxiety in the general population, although this conclusion is limited by the heterogeneity of results and still cannot be generalised to other populations due to the low representativeness of the clinical population in this review.

## 1 Introduction

The study of Delay of Gratification (DG) initially emerged within the field of economics (Kirby, 1997). A recent definition formulates it as a psychological construct that involves the choice of larger rewards in the future over smaller and immediate rewards (Buczny, 2020). This ability of individuals has also been addressed by the constructs of Delay Discounting (DD; Madden et al., 2011) and Temporal Discounting (TD; Ruggeri et al., 2022) which, in opposite way, focus on the subjective devaluation of the first reinforcement when delayed over time. Higher levels of DD or TD result in a greater inclination to select immediate rewards over those that are delayed, even when the latter are of a higher value.

Thereby, under these considerations, expressions of DG imply a greater predilection for the delayed stimulus, the one with the greatest load of reward. This definition draws a clear continuum among DG and DD, showing two poles of the same decision making and behavioural guide.

Reviewing common methods of study in this topic, these discounting constructs have been studied using standardised tests like, for instance, the Monetary Choice Questionnaire (MCQ; Kirby et al., 1999) or 5-trial adjusting delay discounting task (5-ADT; Koffarnus and Bickel, 2014) where subjects are offered the possibility of repeatedly choosing between a smaller and closer reward or a larger and more delayed one. These tests lead to a calculation of the DD by the discount rate (k), indicating the preference for immediate rewards over delayed rewards. The higher the k, the greater the preference.

Thereby, the construct DD is regarded as a parameter related to impulsivity (Levitt et al., 2020; Tschernegg et al., 2015). This impulsivity has previously been proposed as a key feature in a range of psychiatric disorders, extending beyond those traditionally associated with high impulsivity, such as attention deficit hyperactivity disorder, disruptive disorder, impulse control and behavioural disorders, or addictive disorders (Crisp and Grant, 2024). Furthermore, it is associated with other disorders, including borderline personality disorder, mood disorders, and panic disorder with agoraphobia (Crisp and Grant, 2024; Kulacaoglu and Kose, 2018). For this reason, DD has been put forth as a transdiagnostic process for certain psychiatric disorders (Amlung et al., 2019).

The physiological mechanisms underlying discounting for delay are not yet fully elucidated. Nevertheless, a significant relationship has been identified between specific activation patterns in particular regions of the brain (Ustárroz and Grandi, 2016), as well as a correlation between delay discounting and grey matter volume in the orbitofrontal cortex and anterior cingulate cortex, two regions of the brain associated with behavioural self-regulation and decision-making (Li et al., 2019) It has been also described the predictability of the delay discounting behaviour in older adolescents regarding the functional dynamic connection of the amygdala, suggesting a likely involvement of mesolimbic and mesocortical dopamine pathways in adolescents with these circuits already developed (Mertens et al., 2025).

Nevertheless, although the concept and operation seem clear, and the definition sound, some authors have recently pointed out inconsistencies in DD research paradigm (Bailey et al., 2021). Under that discussion, the first challenge concerns the high conceptual heterogeneity found in the literature. This heterogeneity could underscore relevant nuances of the process across samples and situations, and would benefit from a disambiguation of the field.

On the same line, the systematic review and meta-analysis from Weinsztok et al. (2021) highlights the considerable heterogeneity and also discrepancy in results, thereby indicating a substantial impact of publication bias in studies employing smaller samples.

Moreover, Yeh et al. (2021) conducted a study that examined the association between impulsivity, other personality traits and intelligence while controlling for socioeconomic factors, and the findings indicated the likely presence of bidirectional relationship between these socio-economic factors and the capacity to delay gratification. An indirect relation not commonly addressed that warrants further investigation the extend the construct.

Apart from this clarification, and regarding the application to the field of health, DD has been the subject of extensive study in the context of addiction. Individuals with Alcohol use disorder (AUD; American Psychiatric Association, 2022) have been found to show a higher discount rate (k), then a more intense preference for closer reward than healthy controls (Gowin et al., 2019). Also, in individuals who consume both cannabis and alcohol, particularly those with more severe alcohol abuse, exhibit a greater degree of DD than those who solely use alcohol or those with less severe alcohol addiction (MacKillop et al., 2010; Phung et al., 2019). Among users of other substances such as tobacco, heroin or cocaine, they have also been reported with higher scores on discount rates compared to healthy controls (Kirby and Petry, 2004; Konecky and Lawyer, 2015). Even in the fuzzy boundary of addictive substances, it has been found significant relationships between high rates of DD and e-cigarette use, concurrently in size effect to those relations reported in tobacco users (Białaszek et al., 2017). This find suggests a pattern of higher discounting rates across a wide variety of potentially addictive disorders, even including the so-called non-substance addictions (Amlung et al., 2017). Nevertheless, recalling the hitherto explained discrepancies, a recent meta-analytic review on addiction indicated the persistence of substantial heterogeneity in findings, hindering robust conclusions (Weinsztok et al., 2021).

In a broader scope, the DD construct has been the subject of study also in other areas of clinical psychology research. For instance, Pinto et al. (2014) investigated the mechanism of DD in individuals with obsessive-compulsive disorder (OCD) and obsessive-compulsive personality disorder (OCPD). Their findings revealed lower scores on DD tasks in individuals with OCPD, irrespective of whether they also had OCD, when compared to healthy controls and individuals with OCD alone. The authors suggest that these findings are consistent with the characterisation of perfectionism and rigidity in OCD and contribute, on their opinion, to a deeper understanding of this concept in the context of specific disorders.

In their meta-analysis, Amlung et al. (2019) reported results were levels of DD compared to healthy controls were systematically higher in all disorders included, excepting for anorexia nervosa and obsessive-compulsive disorder. This is consistent with the psychological inflexibility and excessive control characteristic of these individuals.

Aiming at these eating disorders, the study carried out by Steward et al. (2017) points out the DD as a potentially fruitful avenue of investigation for enhancing our understanding of eating disorders. The capacity to defer gratification has been examined in patients with anorexia nervosa, both the restrictive and the purging subtypes, as well as those with binge eating disorder, in comparison with healthy controls. As anticipated, the DD tasks revealed higher scores in subjects with binge eating disorder and purge-type anorexia nervosa compared to those with restrictive-type anorexia nervosa (Steward et al., 2017). Additionally, a meta-analysis by Amlung et al. (2016) exposed how individuals with low self-control towards eating stimuli and obesity consistently exhibited a diminished capacity to delay gratification.

Also, in the treatment and monitoring of certain chronic diseases, the capacity to hold up rewards for better achievements has been described as of paramount importance (Conthe et al., 2014). As some reports indicates, a higher k rate is associated with lower engagement in monitoring health behaviours in patients with diabetes (Campbell et al., 2021; Epstein et al., 2021). Furthermore, DD is associated with poor prognosis for psychosocial adjustment, and also with the development of anxiety and depressive symptoms (Anderson and Stanger, 2023; Campbell and Egede, 2023). Conversely, the resistance to palliative care and accepting support services that could greatly improve the well-being of patients with conditions like prostate cancer is puzzling. This reveals an important insight: patients often choose to avoid the immediate effort or discomfort of health-promoting actions, even if it could lead to better well-being in the long run (Gerhart et al., 2016).

The impact of anxiety on public health in recent years appears to be a significant and growing concern (Penninx et al., 2021; Latas et al., 2019), but the current evidence base on the relationship between DD and anxiety is still relatively limited. One illustrating example of this relation is the randomised experimental study conducted by Rounds et al. (2007) subjects with high levels of social anxiety demonstrated significantly greater discounting than subjects with low levels of social anxiety in the non-threat condition. This finding suggests a notable difference in the perceived value of rewards under normal conditions between the two groups. Xia et al. (2017) assessed the impulsivity of subjects with high trait anxiety in comparison to subjects with low trait anxiety through a delay discounting task. The authors postulated intolerance to uncertainty as a potential explanation for the observed results. Other authors have proposed that delay discounting may act as a mediator between psychological inflexibility and various mental health issues, including depression, anxiety, eating disorders and hostility (Levin et al., 2018).

Again, there are other construct validity issues that may be contributing to the emergence of contradictory results across studies. In recent research, Armstrong and Hoge (2024) studied the relationship between DD and the experience of anxiety in patients diagnosed with anxiety disorders, and their results were not significant, contrasting with the previous publications showing a notable correlation between anxiety and impulsivity across diverse populations (Cheng et al., 2022; Guo et al., 2024; Kulacaoglu and Kose, 2018; Moustafa et al., 2017).

Thereby, the considerable variability in the conceptual approach to the DD paradigm, in conjunction with the absence of a robust theoretical model, has the potential to hinder the interpretation of results. The incorporation of studies examining diverse approaches could facilitate the elucidation of the specific relationship between DD and anxiety.

In conclusion, although a wide body of research has been published suggesting a relationship between various psychological disorders and delay discounting (DD), and some authors have tried to correlate DD with personality or intelligence factors, the relationship between DD and anxiety itself remains unclear. This systematic review aims to gather a wide scope set of studies related to DD and anxiety in order to analyse the recent research, conducted over the past 10 years, in both clinical and general populations. This review shall help to assess the current conceptual knowledge and, thereby, to clarify the cross-sectional elements of the construct as well as pointing out the main discrepancies still to solve.

## 2 Methods

A systematic review was conducted in accordance with the PRISMA 2020 guideline (Page et al., 2021).

### 2.1 Search strategy and sources

A systematic search was conducted in PubMed, Web of Science (WoS) and Scopus databases. The search was limited to scientific articles published in peer-reviewed journals, written in English and Spanish. To identify all relevant articles published between 2013 and 2023 the search was conducted using the term related to DG (using the operator “or”) and the addition to the term “anxiety” with the operator “and,” in title, abstract or key words. The variety of terminology employed throughout the literature was chosen with the objective of ensuring inclusion of all potentially pertinent studies in the review. The terms referring DG were (‘discounting’, ‘reward delay’, ‘delay of gratification’, ‘delayed reinforcement’, ‘intertemporal choice’ and ‘intertemporal decision’).

### 2.2 Eligibility criteria

The following criteria was set: Only empirical papers published in peer-reviewed journals and focused on human samples were included. They must show a clear description of definitions and assessments of the constructs of interest, hitherto explained, anxiety and delay of gratification or any of their names, provided a proper definition.

As inclusion criteria, Delay of Gratification was defined as any test in which subjects were presented with the option of receiving a smaller, immediate reward or a larger but delayed reward instead. Additionally, Anxiety measurement was included only if a standardised psychometric test was applied, or a direct expression of the subject was recorded.

Apart from not meeting the inclusion criteria, exclusion criteria included non-pair-review-type papers, defining concepts in terms not related to behavioural research, not including clear definition of constructs of interest, or not explicitly addressing the relation between Gratification Delay and Anxiety.

### 2.3 Selection process

**Figure 1:**
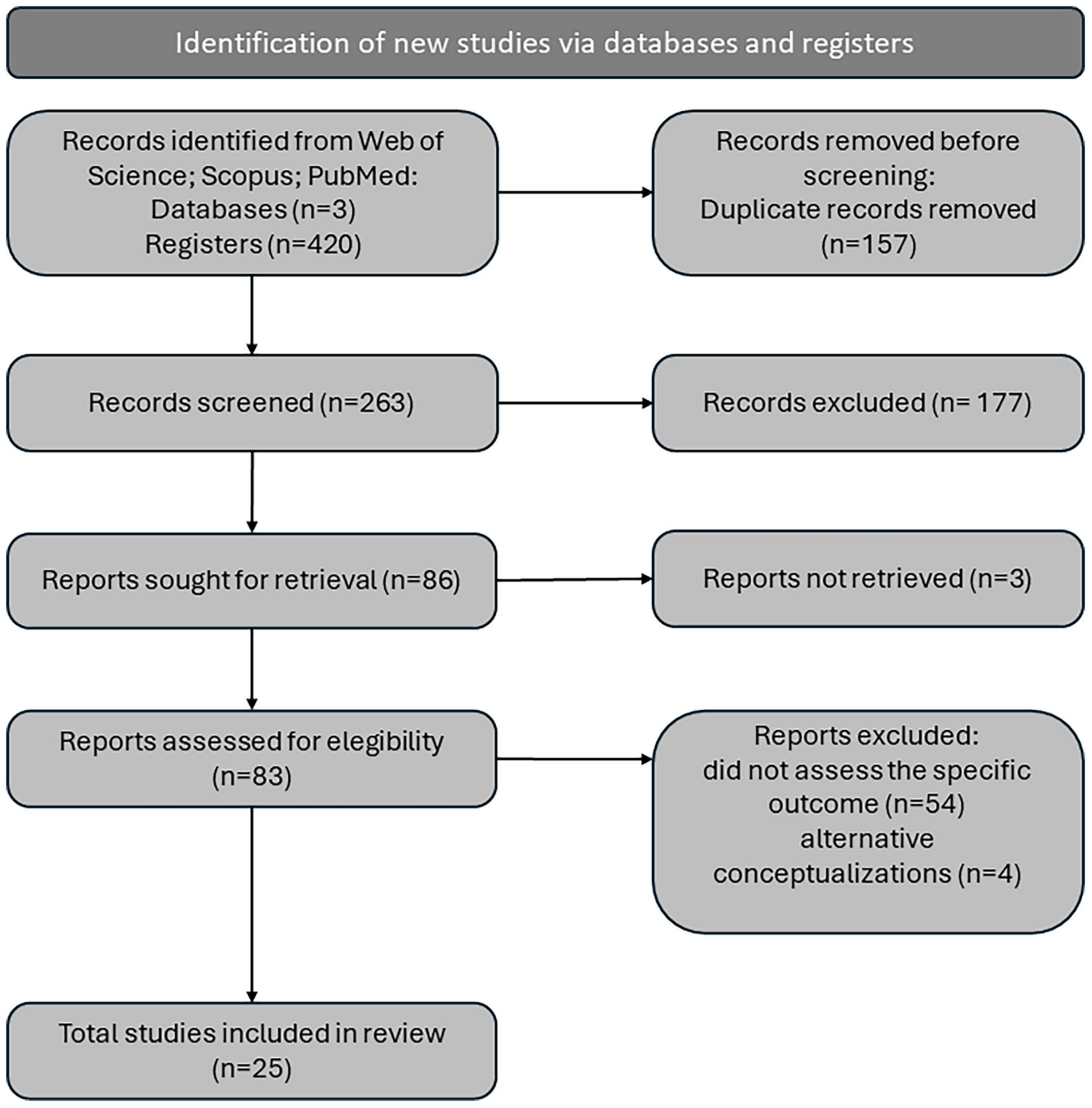
Summary of the PRISMA flow diagram.

The selection of documents for review was conducted by a single reviewer basing on search strategy (Figure 1). The overall review process was supervised by two independent reviewers.

The following terms were considered: ‘delay discounting’ and ‘temporal discounting’, which refer to the devaluation of reinforcement as it is delayed (Amlung et al., 2019); ‘intertemporal choice/decision’ (Kalenscher and Pennartz, 2008; Marchetti et al., 2014) which, in a more general sense, examines the discrepancy in the perceived value of reinforcement contingent on its temporal positioning; and ‘Delay of Gratification’ or ‘Reward Delay’, which underscores an individual’s capacity to defer gratification (Fernández García et al., 2021).

The final search was conducted on 14 January 2024. A total of 205 results were retrieved from the WoS database, 163 from Scopus, and 52 from PubMed. A total of 157 papers were identified as duplicates and subsequently excluded. The remaining 263 abstracts and methods were then reviewed to ascertain whether they met the inclusion criteria. A total of 177 papers were excluded from further analysis, as they did not meet the pre-established criteria. Among excluded reports, 88 were deemed irrelevant to the topic, primarily due to the non-behavioural application of the term ‘discounting’, 46 studies were found to have employed an invalid measure for anxiety, as defined by established criteria, 23 studies did not studied human subjects, 14 were excluded as they differed in design from those for which this review was conducted, four were not scientific articles, and two studies did not assess delay of gratification. The remaining 86 papers were then subjected to a further assessment to determine their eligibility. A further examination of the reports led to the exclusion of 54 papers on the grounds that they did not explicitly assess the relationship between delayed gratification and anxiety, and a further four on the basis that they addressed concepts that were incompatible with the objective of this review. Three articles were excluded from this review due to unavailability of the necessary resources. The remaining 25 articles were subjected to a process of data extraction, the results of which are presented in Table 1.

**Table 1:** Summary of studies included in the review.

Authors	Sample					Design	Anxiety assessment	DD assessment**	Main results
	N	Age (X̅ ± SD)	Detail*	Biol. gender (% Female)	Diagnosis				
Li et al. (2023)	1,417	13.83; 1.48	China	48.3%	No	observational, Cross-sectional	CASI	MCQ	Negative correlation between ICR of MCQ and CASI (not significant)
Levin et al. (2018)	389	20.1; 3.5	Caucasian	69.7%	No	observational, Cross-sectional	CCAPS-34	ADT-5	General anxiety and ADT *r* = −0.11
Zampella and Benau (2022)	152	35.5;11.6	USA	45.0%	No	Observational, Cross-sectional	DAS-E	DGI-35	*r* = −0.50 DAS-E and DGI
Nigro et al. (2017)	1,010	15.37; 2.05	Italian	52.5%	Gambling	Observational, Cross-sectional	DASS-21	MCQ	*r* = 0.077 MCQ and DASS
Jiao et al. (2022)	609	Young adults	Chinese	59.8%	No	Observational, Cross-sectional	GAD-7; PHQ-9	ADOG	*r* = −0.209 GAD and ADOG; −0.186 PHQ and ADOG
Zhang et al. (2020)	105	20.45; 1.87	Chinese	54.3%	No	Observational, Cross-sectional	STAI	Ad hoc design	*r* = 0.34 STAI and discount rate
Haines et al. (2020)	132; 800; 35	20.1; 4.6 and 35.1; 10.8 and 35.8; 10.3	USA	53.6%	Substance abuse	Observational, Cross-sectional	STAI	Ad hoc design	*β* = [−0.045, −0.011] STAI-S and reward valuation
Campbell and Egede (2023)	365	61.8	USA	29.8%	Type 2 diabetes	Observational, Cross-sectional	GAD-8	QDQ	beta = 0.52; delay aversion and anxiety; DD and anxiety beta = 0.46
Steinglass et al. (2017)	119	30;4 and 29;7.6	USA	43% and 48%	SAD; AN; OCD	Observational, Cross-sectional	STAI	Ad hoc design	Discount and STAI-T *r* = −0.210. SAD and discount t (188) = 1.82; *p* = 0.071
Patt et al. (2021)	44	19; 1.2		100.0%	No	Observational, Cross-sectional	STAI	Ad Hoc scale	STAI-S and AUC experiential *β* = −0.33, *p* = 0.049. STAI-T and AUC experiential *β* = 0.48, *p* = 0.003
DeRosa et al. (2023)	1843	10.6; 3.17	Varied	33.8%	9 different	Observational, Cross-sectional	K-SADS	ADT-5	Positive correlation between anxiety and discount rate in food reward measure.
Gerhart et al. (2016)	212	62; 8	Caucasian	0.0%	Prostate Cancer	Observational, Cross-sectional	DASS-21	DGI-10	*r* = −0.23 Anxiety and DG
Xia et al. (2023)	1,016	[17–26]	Chinese	60.9%	No	Observational, Cross-sectional	GAD-7, PHQ-9	ADOG	*p* = −0.238 GAD-7 and ADOG; *p* = −0.189 PHQ-9 and ADOG
Minhas et al. (2020)	730; 602	21.44;1.19 22.63;1.03	Canada USA	52.6% 57.3%	HED	Observational, Cross-sectional	GAD-7, PHQ-9	ADT-5	Higher discount rate in high and moderate psychiatric severity groups.
Levitt et al. (2023)	1,388	38.99; 13.70	Canada *	57.9%	No	Observacional, Cross-sectional	PHQ; PHQ-Anx	MCQ	*r* = 0.15 high discount rate and PHQ; *r* = 0.1 high discount rate and PHQ-anx
Morasco et al. (2019)	75				Chronic pain	Observacional, Cross-sectional			Positive correlation between discount rate and anxiety.
Robertson et al. (2023)	202	Adults	UK	50.9%	No	Observacional, Cross-sectional	DASS-21	Ad hoc design	Not significant between discount rate and anxiety 𝐶𝐼90% = [−0.01, 0.1]
Ho et al. (2023)	414	15.6; 0.6	Vietnamese	49.0%	No	Observacional, Longitudinal	DASS-21	ADT-5	Discount at T1 predicted anxiety at T2 *β* = −0.10,
Worthy et al. (2014)	56	Young adults	USA		No	Experimental, Cross-sectional	BAI	Ad hoc design	Anxiety and proportion of times participants selected the increasing option throughout the task *r* = − 0.12 not significant
Xia et al. (2017)	52	19.4 and 19.7	Chinese	50.0%	No	Experimental, Cross-sectional	STAI-T	Ad hoc design	ANOVA [*F* (1, 50) = 4.75, *p* = 0.034, η^2^p = 0.087] Impulsivity within-subject factor and anxiety between subject factor.
Tanovic et al. (2018)	54	21.2;2.59	Caucasian 57.40%	72.2%	No	Experimental, Cross-sectional	Likert 7	TCIP and MCQ	TCIP and anxiety *r* = −0.03; MCQ and anxiety *r* = −0.31 Not significant
Zhao et al. (2015)	108	19.28	Caucasian	61.0%	No	Experimental, Cross-sectional	STAI	MCQ	Anxiety trait x state *β* = −0.222 *p* = 0.034
Hurlemann et al. (2019)	70	31.21; 11.43 and 34.46; 14.45	Netherlands	80.0%	SAD	Experimental, Cross-sectional	SPAI	Ad hoc design	Higher discount rate in SAD group vs. control.
Jenks and Lawyer (2015)	113	25.1; 8.2 and 26.4; 8.8	Caucasian	67.0%	SAD	Experimental, Cross-sectional	SIAS and Subjective measure	Ad hoc design	The induction of anxiety did not result in a higher discount rate
Gui et al. (2023)	439	Adults	Chinese	57.5%	No	Varied	STAI	Ad hoc design	*r* = −0.581 between state anxiety and decision weight on MoneyDiff

*Features reported differed in country and ethnics among publications. **CASI, Childhood Anxiety Sensitivity Index; MCQ, Monetary choice Questionnaire; STAI, State Trait Anxiety Inventory; CCAPS, Counselling Centre Assessment of Psychological Symptoms; DASS, Depression Anxiety and Stress Scale; K-SADS, Kiddie Schedule for Affective Disorders and Schizophrenia; ADT-5, 5-Trial Adjusting Delay Discounting Task; BAI, Beck Anxiety Inventory; DGI, Delaying Gratification Inventory; ADOG, Academic Delay of Gratification; TCIP, Two-Choice Impulsivity Paradigm; PHQ-9, Patient Health Questionnaire; PHQ-Anx, Patient Health Questionnaire Anxiety Subscale; SPAI, Social Phobia and Anxiety Inventory; GAD, Generalised Anxiety Disorder Scale; DAS-E, 53-item extended Death Anxiety Scale; QDQ, Quick Delay Questionnaire; SIAS, Social Interaction Anxiety Scale; SAD, Social anxiety disorder; HED, Heavy episodic drinking; AN, Anorexia nervosa; OCD, Obsessive-compulsive disorder.

### 2.4 Data collection process

The extracted data included the following characteristics: sample size, age and standard deviation, nationality, sex ratio, and diagnosis; study type; measure of anxiety; measure of delay discounting; and main outcomes of interest.

## 3 Results

### 3.1 Study type

The present review identified 18 studies that employed an observational methodology, six with experimental designs, and a last one of a mixed methods approach.

Of the 25 studies included, only one employed a longitudinal methodology, while the remainder employed a cross-sectional approach. Anxiety was assessed using a variety of instruments, with the most frequently employed being the State–Trait Anxiety Inventory (7; Spielberger et al., 1983) the Depression Anxiety and Stress Scale (4; Lovibond and Lovibond, 1995), the Generalised Anxiety Disorder Scale (4; Johnson et al., 2019), and the Patient Health Questionnaire (4; Arroll et al., 2010). With regard to the assessment of DG, an *Ad Hoc* design was utilised in the majority of studies (10), followed by the Monetary Choice Questionnaire (5; Kirby and Maraković, 1996) and the Adjusting Delay Discounting Task (4; Koffarnus and Bickel, 2014).

### 3.2 Sample features

The total number of subjects included in the 25 studies reviewed was 12,728, with an age range of 13 to 69 years and a sex ratio that varied from 0 to 100% female. The samples encompass a diverse range of nationalities, with the most prevalent being the United States and China (Campbell and Egede, 2023; Haines et al., 2020; Jiao et al., 2022; Li et al., 2023; Minhas et al., 2020; Steinglass et al., 2017; Worthy et al., 2014; Xia et al., 2017, 2023; Zampella and Benau, 2022; Zhang et al., 2020) followed by Italy, the United Kingdom, and Vietnam (Ho et al., 2023; Nigro et al., 2017; Robertson et al., 2023).

The ethnical consideration of subjects varied widely among studies. Three of them (Gui et al., 2023; Hurlemann et al., 2019; Levitt et al., 2023) only specified the geographical origin of the sample, rather than nationality or ethnicity, while others chose to specify the ethnic background of the sample- Within that latter type, the majority of subjects were Caucasian, although there were also smaller percentages of Hispanic, Asian, and African American populations (DeRosa et al., 2023; Gerhart et al., 2016; Jenks and Lawyer, 2015; Levin et al., 2018; Tanovic et al., 2018; Zhao et al., 2015). Finally, two studies did not report this information (Morasco et al., 2019; Patt et al., 2021).

Lastly, regarding the clinical aspect of samples, a total of 15 samples were drawn from the general population (Gui et al., 2023; Ho et al., 2023, p. 23; Jiao et al., 2022; Levin et al., 2018; Levitt et al., 2023; Li et al., 2023, p. 23; Patt et al., 2021; Robertson et al., 2023; Tanovic et al., 2018; Worthy et al., 2014; Xia et al., 2017, 2023; Zampella and Benau, 2022; Zhang et al., 2020; Zhao et al., 2015), and only nine reports were included focusing on clinical or subclinical samples with various pathologies (Campbell and Egede, 2023; DeRosa et al., 2023; Gerhart et al., 2016; Haines et al., 2020; Hurlemann et al., 2019; Jenks and Lawyer, 2015; Minhas et al., 2020; Morasco et al., 2019; Nigro et al., 2017; Steinglass et al., 2017).

### 3.3 Anxiety and DD

Of the studies reviewed, 16 provided evidence in favour of the existence of a positive association between anxiety and DD. This evidence suggests that higher levels of anxiety are related to higher discount rate, which means a lower ability of subjects to delay gratification. Significant associations supporting this hypothesis were identified in 12 of the papers (Campbell and Egede, 2023; Gerhart et al., 2016; Ho et al., 2023; Jiao et al., 2022; Levin et al., 2018; Levitt et al., 2023; Morasco et al., 2019; Nigro et al., 2017; Xia et al., 2017, 2023, p. 23; Zampella and Benau, 2022; Zhang et al., 2020), Two studies did not provide the corresponding statistical analysis (Hurlemann et al., 2019; Minhas et al., 2020) while two others reported non-significant associations (Tanovic et al., 2018; Worthy et al., 2014).

Two of the studies reviewed yielded evidence that contradicts the hypothesis proposed in this study. In contrast with the hypothesis proposed in this study, Li et al. (2023) discovered negative, albeit non-significant, correlations between Immediate Choice Ratios (ICR) calculated from the MCQ test and anxiety sensitivity obtained from the Childhood Anxiety Sensitivity Index instrument. This suggests that as anxiety sensitivity increases, the percentage of times the immediate option is selected decreases. Conversely, Jenks and Lawyer (2015) observed no significant increase in the discount rate in either the control or experimental group when anxiety was induced through public speaking in subjects with social anxiety disorder.

With regard to the remaining articles under review, the results are inconclusive (DeRosa et al., 2023). Furthermore, the interactions between factors are complex and require further elucidation. Haines et al. (2020) observed that high state anxiety scores were a reliable predictor of a devaluation of reinforcement in participants. Moreover, anxiety was demonstrated to act as a robust mediator between impulsivity and discounting rates, rather than exerting a direct influence on discounting rates. A statistical analysis of all participants in the Steinglass et al. (2017) study revealed a negative correlation between discounting rates and anxiety. However, the group with a diagnosis of Social Anxiety Disorder exhibited higher delay discounting rates than healthy controls. In contrast, the experimental design proposed by Patt et al. (2021) to investigate intertemporal choices across an experiential and a hypothetical task revealed disparate associations between state anxiety and trait anxiety with these tasks. Specifically, state anxiety was found to be positively associated with the discount rate in the experiential task, while no correlation was observed between the discount rates of the experiential and hypothetical tasks.

The study by Robertson et al. (2023) revealed no correlation between anxiety and discount rate. However, the researchers did identify a relationship between anxiety and temporal representation through language. In contrast, the study by Gui et al. (2023) employed the Drift Diffusion Model (DDM) to investigate delay discounting. Their findings indicated a significant correlation between state anxiety and the weight assigned to monetary differences in decision-making. This suggests that individuals with high state anxiety may devalue monetary differences and exhibit increased impulsivity.

In the study by Zhao et al. (2015) a significant interaction between state and trait anxiety was observed. Individuals with high state and trait anxiety exhibited a tendency to choose delayed rewards, whereas those with high trait anxiety but low state anxiety demonstrated a preference for more immediate rewards.

### 3.4 Risk of bias

The demographic characteristics of some studies compromise the external validity of the results. Of the 25 studies reviewed, 6 did not reach a sample size of 100 (Hurlemann et al., 2019; Morasco et al., 2019; Patt et al., 2021; Tanovic et al., 2018; Worthy et al., 2014; Xia et al., 2017) and 4 only slightly exceeded it (Jenks and Lawyer, 2015; Steinglass et al., 2017; Zhang et al., 2020; Zhao et al., 2015) which could jeopardize their representativeness.

A mere six of the papers subjected to review opted for an experimental design (Hurlemann et al., 2019; Jenks and Lawyer, 2015; Tanovic et al., 2018; Worthy et al., 2014; Xia et al., 2017; Zhao et al., 2015). The interpretation of results in observational studies is susceptible to bias due to the lack of control over variables. Additionally, the six papers included in this review have relatively small sample sizes, which could introduce further bias into the overall results.

One of the characteristics extracted from the studies presented in Table 1 is the diagnosis of the sample. It should be noted, however, that two of the nine studies with clinical samples did not carry out an actual diagnostic process of the pathology (Jenks and Lawyer, 2015; Nigro et al., 2017). Rather, the sample was selected or assigned to the control or experimental group based on the scores obtained in validated questionnaires. The remaining studies reviewed did, however, carry out this process (Campbell and Egede, 2023; DeRosa et al., 2023; Gerhart et al., 2016; Haines et al., 2020; Hurlemann et al., 2019; Morasco et al., 2019; Steinglass et al., 2017). The aforementioned variability in sample selection may result in the drawing of conclusions regarding the population in question that are biased in one way or another.

The incorporation of a control group is a crucial element in research, as it enhances the reliability and validity of the findings. A review of the 24 studies revealed that 14 of them lacked a control group, thereby preventing a comparison of the results of their observations (DeRosa et al., 2023; Gerhart et al., 2016; Ho et al., 2023; Levin et al., 2018; Levitt et al., 2023; Li et al., 2023; Nigro et al., 2017; Patt et al., 2021; Tanovic et al., 2018; Worthy et al., 2014; Xia et al., 2017, 2023; Zampella and Benau, 2022; Zhang et al., 2020). Although the majority of the studies were conducted in the general population, the inclusion of a comparative group outside the study population could have been beneficial.

A significant source of bias is the considerable heterogeneity in the assessment of both DD and anxiety across the reviewed studies. On the one hand, the majority of studies employ an *ad hoc* design for the assessment of DD. For the remaining studies, only two tests are utilised by more than one study. While the fundamental paradigm of the tests is consistent, the specific characteristics vary, which may influence the results, as illustrated in the study by Patt et al. (2021). A similar phenomenon is observed in the anxiety measures. Despite the use of standardised measures in all but one study (Tanovic et al., 2018) the variability in measurement methods considerably reduces the reliability of the results.

## 4 Discussion

The operational objectives of this review were to examine the recent results on correlation between delay discounting and anxiety, and secondly, to actualise and disambiguate the underlying mechanisms that may be responsible for this association.

As hitherto explained, the relationship between subjects with social anxiety has previously been evaluated with contradictory results among different research studies (Armstrong and Hoge, 2024; Rounds et al., 2007) and the underlaying mechanism is yet to fully discover (Li et al., 2019; Mertens et al., 2025; Ustárroz and Grandi, 2016).

A recent study conducted in Canada with a considerably larger sample size identified more pronounced discount rates among subjects diagnosed with Social Anxiety Disorder, Post-Traumatic Stress Disorder, and Generalised Anxiety Disorder when compared to healthy controls. However, these differences were not replicated in subjects diagnosed with Panic Disorder and Obsessive-Compulsive Disorder (Boyd et al., 2024). These results align with those of other studies, indicating that the interplay between anxiety and DD may not follow a uniform pattern across all disorders (Amlung et al., 2019; Steinglass et al., 2017). This suggests that even within the domain of anxiety disorders, the association between these two phenomena may not be consistent.

### 4.1 Anxiety and DD in general population

A lower DD rate among low-anxious versus high-anxious subjects has been found in 10 out of 15 general population studies (Ho et al., 2023, p. 23; Jiao et al., 2022; Levin et al., 2018; Levitt et al., 2023; Tanovic et al., 2018; Worthy et al., 2014; Xia et al., 2017, 2023; Zampella and Benau, 2022; Zhang et al., 2020). These studies provide evidence in favour of the existence of a positive relationship between anxiety and DD. Furthermore, additional noteworthy findings pertaining to this relationship have been identified in four of the remaining five articles (Gui et al., 2023; Patt et al., 2021; Robertson et al., 2023; Zhao et al., 2015). While these findings do not directly indicate a direct correlation between DD and anxiety, they do contribute valuable insights that enhance the comprehension of this relationship. In relation to the general population, the evidence presented in this review is sufficient to affirm that there is a positive association between DD and anxiety in people with gambling disorder. In other words, elevated anxiety levels are associated with reduced capacity to delay gratification in individuals, a phenomenon that appears to be generalisable across different ethnicities and nationalities, as well as to both sexes. This effect is observed consistently across studies, despite the wide range of anxiety and DD measures employed. These findings provide preliminary evidence for this relationship in the general population, although further research is needed to substantiate these observations.

### 4.2 Anxiety and DD through medical illnesses

The three studies on medical illnesses included in this review indicate that anxiety is positive related to DD among these subjects. This finding is analogous to that observed in the general population sample. These results align with those reported in previous research (Craft et al., 2020; Epstein et al., 2021). They suggest that this construct is a fundamental aspect to be considered in the development of more effective intervention protocols that take into account aspects of patients’ psychology.

### 4.3 Anxiety and DD in addictions

The close relationship between delay discounting and patients with various addictions is well established in the literature (MacKillop et al., 2011; Weinsztok et al., 2021). Nevertheless, further research is required to substantiate the existence of an altered pattern of delay discounting that is a common feature of all addictive disorders. Furthermore, it would be beneficial to incorporate a range of terminologies beyond DD to ensure the inclusion of diverse approaches to the study of this paradigm. The DD paradigm has facilitated a more nuanced understanding of addictive mechanisms in both substance and non-substance addictions. The direction of this relationship is not yet entirely clear, although this is beyond the scope of this review. However, the results of the three studies included in this review related to addictions are in line with studies specific to this subject (Amlung et al., 2017; Konecky and Lawyer, 2015).

### 4.4 Anxiety and DD in social anxiety disorder

The study of the role of DD among people with social anxiety disorder has yielded conflicting evidence (Armstrong and Hoge, 2024; Boyd et al., 2024). In our analysis, two of the three studies have indicated a positive correlation between SAD and DD. Both Hurlemann et al. (2019) and Steinglass et al. (2017) identified a positive correlation between a SAD diagnosis and a higher discounting rate. This indicates that individuals with this diagnosis discount delayed reinforcement more strongly, thereby demonstrating a preference for immediate and smaller rewards. Conversely, Jenks and Lawyer (2015) employed an experimental design in which anxiety was induced through a public speaking task and observed no variation in discounting rates in either the experimental group with this diagnosis or the control group. A synthesis of these findings suggests that the increase in the discount rate may not be a consequence of anxiety, but rather a potential causal factor. While correlational studies offer valuable insights into a phenomenon, experimental designs are essential for elucidating the underlying mechanisms of observed correlations.

### 4.5 DD and trait–state anxiety

The interaction between state and trait anxiety (Zhao et al., 2015) offers an intriguing perspective on the role of DD in anxiety experience. Therefore, individuals with high trait anxiety may be more susceptible to exhibiting a risk perception bias for delayed rewards during periods of elevated anxiety compared to periods of low anxiety. Consequently, they may opt for immediate, albeit modest rewards due to the fear of missing out on larger, albeit more delayed rewards. Those with low trait anxiety are less susceptible to this bias, demonstrating superior performance even in high-anxiety contexts (Kim et al., 2020; Notebaert et al., 2016). Haines et al. (2020) propose that there may be an interaction between individuals’ experienced anxiety and their impulsivity-trait, with the latter influencing the speed at which subjects discount rewards. This implies that the devaluation of reinforcement shown by individuals with high state anxiety could produce more random responses in the DD paradigm, which would limit the validity of the results.

### 4.6 Temporal representation, anxiety and DD

Conversely, it is conceivable that individuals with elevated anxiety levels may exhibit heightened discounting tendencies, not directly, but through a more distanced representation of the future that mediates the experience of anxiety and DD (Robertson et al., 2023). The role of temporal representation as a mediator requires further investigation in future studies. The direction of causality in this relationship, namely whether anxiety produces this temporal distortion or whether it is the temporal distortion that influences the development and experience of anxiety, remains unclear. Furthermore, this relationship has been studied through the lens of language. While the relationship between the use of verbal forms and decision-making is assumed (Banerjee and Urminsky, 2022), it would be beneficial to explore other paradigms.

### 4.7 Anxiety and drift diffusion model

An alternative and more recent approach to the delay discounting paradigm is the Drift Diffusion Model (DDM), which integrates a greater number of variables than the traditional DD paradigm. The traditional DD paradigm employs the parameter k (discount rate) to evaluate this process. However, this parameter does not adequately reflect the complexity of the trade-off between delay and magnitude of reinforcement. In a study conducted by Gui et al. (2023) the influence of smartphone addiction and anxiety on impulsive decision-making was investigated through the lens of the DDM. The findings revealed a significant correlation between monetary differences in decision weight and anxiety, but not between anxiety and the difference in reward delay. These findings provide further insight into the cognitive mechanisms underlying the intertemporal decision process, indicating substantial variability between individuals with high and low state anxiety with respect to the balance they consider optimal for making a decision between immediate and small versus delayed and larger rewards. That is, the temporal delay of these rewards is of lesser importance than the magnitude of the delayed reward, such that a larger magnitude increase in reinforcement is required to compensate for the delay.

### 4.8 Test features and discount rates

In the course of this review, a multitude of methodologies for measuring DD have been employed, all of which are predicated on the dichotomy between immediate, albeit modest, rewards and those that are delayed and of a greater magnitude. However, the specific characteristics of the test appear to be relevant to the resulting outcome. The evidence suggests that individuals’ performance on tasks varies depending on whether they are behavioural or hypothetical tasks (Patt et al., 2021). The studies included in this review employ both types of tasks; however, the majority are hypothetical. This could indicate that participants do not demonstrate their true delay of gratification ability in hypothetical tasks due to a lack of real motivational incentives that they otherwise encounter in experiential paradigms or in their own daily lives. Other possibilities could be the overestimation of one’s own capabilities in hypothetical situations or the emotional influence of real situations on decision-making.

## 5 Conclusion

The role of reinforcers has been the subject of extensive study within the field of addiction. It is perhaps unsurprising that this concept of delay discounting has been the subject of extensive exploration and development within this context. However, reinforcement processes are fundamental to understanding any pathology, from mood or anxiety disorders to sexual or eating disorders. Therefore, it seems worthwhile to consider the capacity of patients with such pathologies to delay gratification in order to gain insight into their relationship with reinforcers in their lives. In general, it appears that individuals with anxiety disorders exhibit a reduced capacity to delay gratification, although the precise nature of this phenomenon remains unclear.

Firstly, there is a need for further research utilising experimental and longitudinal designs with the objective of providing insight into the direction of causality between anxiety and DD. Secondly, further studies, such as that conducted by Murray et al. (2018) are required in both adult, child and adolescent populations. These studies should analyse the lifelong stability of DD, as well as its modifiability through specific techniques. Finally, the potential existence of mediators between anxiety and DD should be evaluated in order to facilitate a more precise understanding of this relationship. Some authors (Greenhow et al., 2015; Tanovic et al., 2018) have put forth the notion of intolerance to uncertainty as a potential mediator. Conversely, other authors (Robertson et al., 2023) have proposed temporal representation as a possible mechanism. Worthy et al. (2014) have posited that it is specifically worry, rather than anxiety, that is directly related to DD. Nevertheless, further research in this area is warranted.

The majority of studies of this review have identified a positive correlation between DD and anxiety. This indicates that individuals with elevated anxiety levels tend to exhibit reduced capacity to delay gratification. In other words, those with heightened anxiety tend to prioritise immediate, albeit smaller, rewards over larger, delayed rewards. The sample size of this review is insufficient to permit the same confidence in this claim for the clinical population. However, the results obtained suggest that people with a psychological disorder or people undergoing treatment for a medical illness could also follow the same pattern, except in people with some pathologies such as obsessive-compulsive disorder or anorexia nervosa.

## 6 Limitations

It is important to acknowledge the limitations of this review. It is recommended that the conclusions drawn from the results be considered with caution. Given the considerable heterogeneity of the measures identified and the dearth of research on this topic in the existing literature, it was not feasible to conduct a meta-analysis of the results. It is recommended that future research include stricter article selection criteria to make meta-analysis possible. However, additional research with observational and experimental designs should also be conducted to contribute to the existing literature on the relationship between anxiety and DD. Furthermore, it is generally advised that at least two independent reviewers be engaged to conduct the screening, selection and eligibility process separately. The research resources available for the present study necessitated the process to be carried out by a single reviewer.

Secondly, although it can be concluded that there is a positive association between DD and anxiety in the general population, the specific mechanisms underlying this association remain to be explored and are beyond the scope of this study.

Thirdly, it has not been feasible to incorporate a sufficient range and quantity of articles examining a diverse array of pathologies into this review to substantiate a definitive conclusion regarding the relationship between anxiety and DD in the clinical population. While the available evidence suggests a potential association, further research with higher methodological standards is required to confirm this.

Fourthly, the extensive range of measurement instruments employed in the studies reviewed represents a significant limitation. Some of the instruments employed, such as the Childhood Anxiety Sensitivity Index, do not specifically assess the experience of anxiety. Furthermore, the Death Anxiety Scale and the Social Interaction Anxiety Scale focus on the experience of anxiety in specific contexts or situations. The measures of these instruments are not an accurate reflection of the experience of anxiety in the moment, which is the purpose of this research. Furthermore, there is even greater heterogeneity in the measures used to assess DD. In future reviews, when research in this field is more prolific, it would be beneficial to restrict the selection of items to those using the same measure.

## Data Availability

The original contributions presented in the study are included in the article/supplementary material, further inquiries can be directed to the corresponding author.
